# Supplementary material for: Influence of Chitin Nanocrystals on the Crystallinity and Mechanical Properties of Poly(hydroxybutyrate) Biopolymer
Source: Polymers (Basel). 2022 Jan 29;14(3):562. doi: 10.3390/polym14030562 (PMC8840629; doi:10.3390/polym14030562)
Supplement: Supplementary file 1 [file polymers-14-00562-s001.zip › polymers-1551199-supplementary.pdf]

Supplementary Information

Influence of Chitin Nanocrystals on the Crystallinity and Mechanical Properties of Poly(hydroxybutyrate) Biopolymer

Marta Zaccone [1,*], Mitul Kumar Patel [2], Laurens De Brauwer [3], Rakesh Nair [3], Maria Luana Montalbano [1], Marco Monti [1] and Kristiina Oksman [2,4,5,*]

1. Proplast, Via Roberto di Ferro 86, 15122 Alessandria, Italy; luana.montalbano@proplast.it (M.L.M.); marco.monti@proplast.it (M.M.)
2. Division of Materials Science, Department on Engineering Sciences and Mathematics, Luleå University of Technology, SE 97187 Luleå, Sweden; mitul.kumar.patel@ltu.se
3. Biobased Europe Pilot Plant (BBEPP), Rodenhuizekaai 1, 9042 Gent, Belgium; laurens.de.brauwer@bbeu.org (L.D.B.); rakesh.nair@bbeu.org (R.N.)
4. Mechanical & Industrial Engineering, University of Toronto, Toronto, ON M5S 3BS, Canada
5. Wallenberg Wood Science Center (WWSC), Luleå University of Technology, SE 97187 Luleå, Sweden

* Correspondence: marta.zaccone@proplast.it (M.Z.); kristiina.oksman@ltu.se (K.O.)

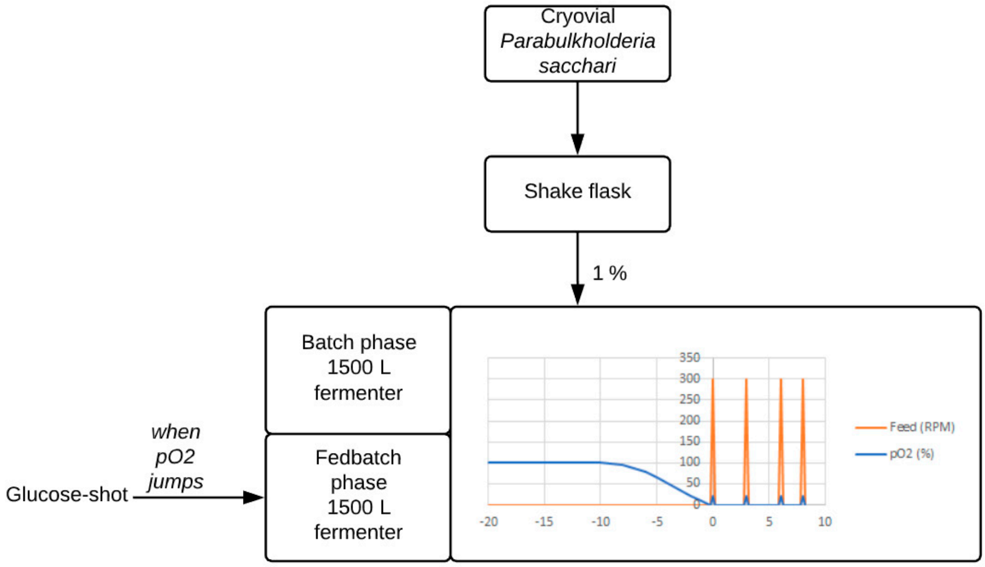

Figure S1. Fermentation process flow diagram for PHB production.

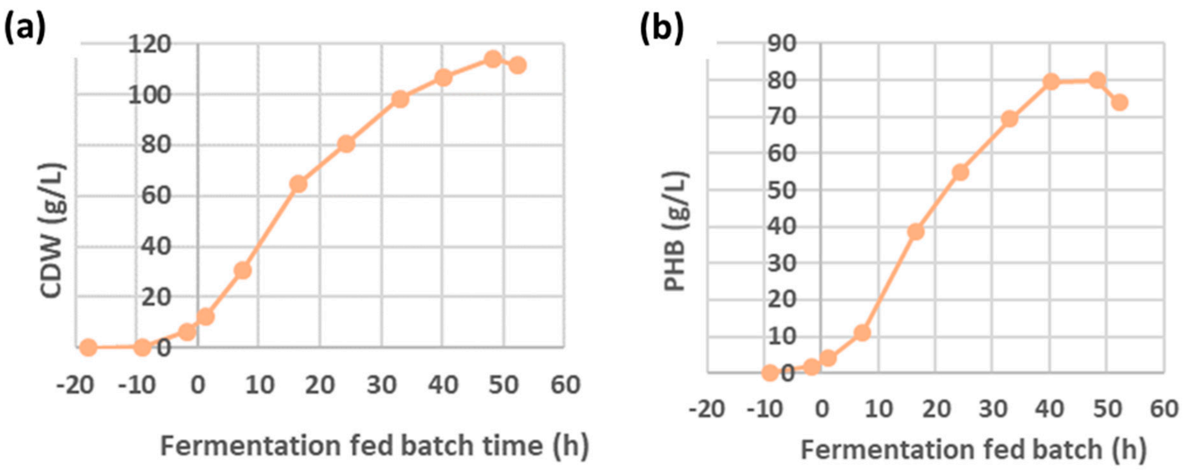

Figure S2. Time course of (a)CDW (g/L) accumulation in a 1.5 m^3 reactor and (b)PHB (g/L) production.

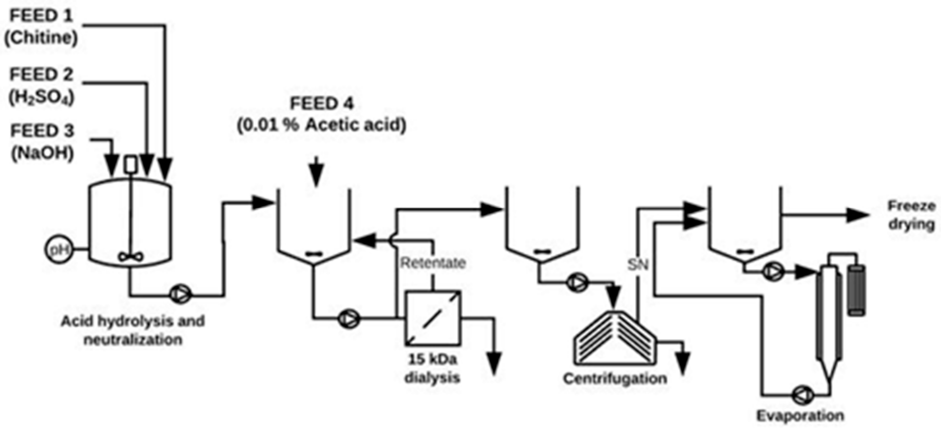

Figure S3. Processing scheme of the large-scale ChNCs **production** process.

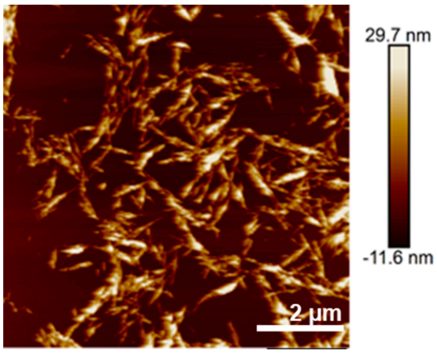

Figure S4. AFM height image of the produced ChNCs showing diameters between 5-15 nanometers, and length between 200-480 nm.

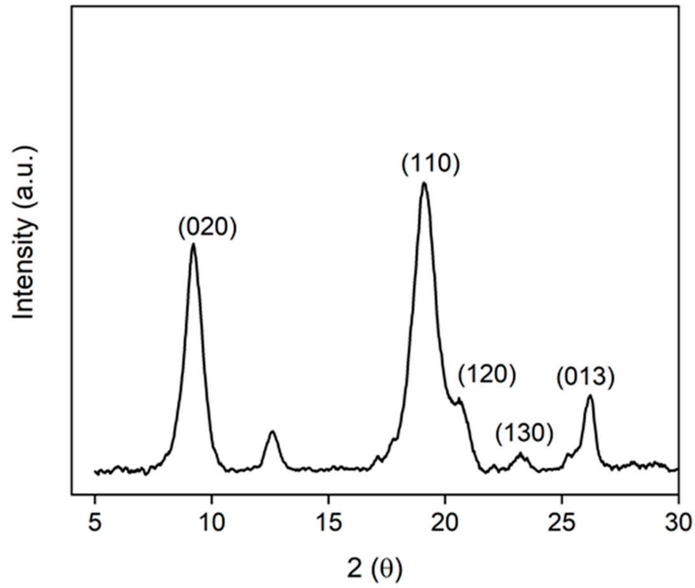

Figure S5. XRD analysis of raw chitin powder confirming the presence of α-crystallite structure of chitin.
